# Analysis of the Spatial Variation of Hospitalization Admissions for Hypertension Disease in Shenzhen, China

**DOI:** 10.3390/ijerph110100713

**Published:** 2014-01-03

**Authors:** Zhensheng Wang, Qingyun Du, Shi Liang, Ke Nie, De-nan Lin, Yan Chen, Jia-jia Li

**Affiliations:** 1 School of Resource and Environmental Science, Wuhan University, 129 Luoyu Road, Wuhan 430079, China; E-Mails: wangzhens@whu.edu.cn (Z.W.); nieke@whu.edu.cn (K.N.); 2 Key Laboratory of GIS, Ministry of Education, Wuhan University, 129 Luoyu Road, Wuhan 430079, China; 3 Shenzhen Center for Health Information, Renmin Road North 2210, Luohu District, Shenzhen 518001, China; E-Mails: ldn308@163.com (D.L.); chy@newhealth.com.cn (Y.C.); jiajiali831@gmail.com (J.L.)

**Keywords:** hypertension, Hierarchical Bayesian models, spatial scan statistics, analysis scale, Shenzhen, urban China

## Abstract

In China, awareness about hypertension, the treatment rate and the control rate are low compared to developed countries, even though China’s aging population has grown, especially in those areas with a high degree of urbanization. However, limited epidemiological studies have attempted to describe the spatial variation of the geo-referenced data on hypertension disease over an urban area of China. In this study, we applied hierarchical Bayesian models to explore the spatial heterogeneity of the relative risk for hypertension admissions throughout Shenzhen in 2011. The final model specification includes an intercept and spatial components (structured and unstructured). Although the road density could be used as a covariate in modeling, it is an indirect factor on the relative risk. In addition, spatial scan statistics and spatial analysis were utilized to identify the spatial pattern and to map the clusters. The results showed that the relative risk for hospital admission for hypertension has high-value clusters in the south and southeastern Shenzhen. This study aimed to identify some specific regions with high relative risk, and this information is useful for the health administrators. Further research should address more-detailed data collection and an explanation of the spatial patterns.

## 1. Introduction

Hypertension is a chronic medical condition in which the blood pressure in the arteries is elevated, and this condition is classified into two categories: primary hypertension and secondary hypertension. Between 90% and 95% of cases are categorized as primary hypertension, which implies high blood pressure with no obvious underlying medical causes [1]. The World Health Organization has identified hypertension as the leading cause of cardiovascular and cerebrovascular mortality and the World’s most common chronic disease, as hypertension is a major risk factor for strokes, myocardial infarctions, heart failures and arterial disease. The treatment of hypertension and its complicating diseases leads to heavy consumption of medical and social resources. The American Heart Association estimated that the projected total costs of high blood pressure will be $91.4 billion in 2015 [2]. There are many risk factors for hypertension disease, including age, race, family history, being overweight or obese, not being physically active, using tobacco, high-salt diet, too little vitamin D and potassium in diet, excessive alcohol use, stress and certain chronic conditions [3,4]. Besides, the risk of having hypertension can vary in regions depending on their environmental conditions and socioeconomic position [5,6].

In China, the prevalence of hypertension has continuously increased during the past fifty years. According to the 2010 Chinese guidelines for the management of hypertension, from 1991 to 2002, the awareness of hypertension increased from 26.3% to 30.2%, the treatment rate rose from 12.1% to 24.7% and the control rate grew from 2.8% to 6.1% [7]. However, these rates are relatively low compared to developed countries. This report reveals that over 130 million of people with hypertension are unaware of their condition and that at least 30 million people are aware of their hypertension but do not receive any medical treatment. Indeed, over 75% of people who are aware that they have hypertension do not adequately control it [7].

Recent hypertension studies on China mainly focus on lifestyle modifications, prevention, the impact on health-related quality of life and medical treatment [8,9,10,11]. Limited epidemiological studies have attempted to describe the spatial variation of the hypertension disease over a large area. Previous works have indicated that neighborhood walkability, food availability, safety, and social cohesion may be mechanisms that link neighborhoods to hypertension [12] and the number of hypertension admission patients largely depends on the general hypertensive population. In this study, we attempted to explore the spatial variation of the hospital admissions for hypertension throughout Shenzhen in 2011. The classic statistic in epidemiology is the standardized ratio (SR) which is commonly used to represent disease risk across a geographical area [13,14] in order to identify those regions with higher or lower disease risk, being useful for capture regional changes. For each region, the standardized ratio is expressed as a relative value between the number of observed cases and the number of expected cases, as estimated by the national disease rate, with or without adjustment for socioeconomic and demographic variables [15].

Although the standardized ratio is a useful tool in disease mapping research, it has some problems. An inevitable problem is spatial autocorrelation, which is an idea often attributed to geographer Waldo Tobler. Measuring the spatial pattern of feature values is based on the notion that things that are near to each other are more alike than things that are far apart. In geographical research, the study area is often delineated by artificial boundaries for measurement or administrative purposes [16]. However, a spatial process in an area has an interaction with neighbors outside these boundaries and adjacent areas usually have similar attributes. In addition, the standardized ratio is a deficient estimator because it depends greatly on the population of each area. Usually, sparsely populated areas with few (or zero) cases can generate extreme values [13,15]. Because the administrative divisions depend on population size, sparsely populated areas are often larger than densely populated areas, and furthermore, they tend to dominate the map visually even though they produce the least precise risk estimates [13]. Moreover, shortcomings in the census data can generate defective risk estimation. For example, rapid population growth since the previous census would cause overestimated risks in a study area [17].

To tackle the spatial dependence and inaccurate estimation of the standardized ratios, many methods have been employed to describe and assess the amount of true spatial variation of disease risk [15]. A disease outbreak can be considered as a geographic process that is highly correlated to a specific geographic location and the corresponding conditions. In GIScience, we analyze the geographic processes for two reasons: first, we seek to predict the likelihood that something will occur in a place [18,19,20]; second, we wish to identify the underlying factors [21,22]. The relationships between various attributes of the spatial data can be defined as a model, which could become quite complex and time-consuming. A Bayesian estimation approach was used to analyze small area diabetes prevalence in the US [23]. For this study, we employ a model-based relative risk estimation method based on hierarchical Bayesian models to assess the true spatial heterogeneity of the relative risk for hypertension admissions. These models are widely used for risk smoothing in disease mapping and have been described in detail by previous works [24,25,26]. The basic principle of Bayesian methods is that uncertain data can be strengthened by combining them with prior information [14]. Such estimates are a compromise between the local value of the standardized ratio and either the mean value for the map as a whole, or some local mean [13]. The distribution for the spatial components in these models is discussed in [27]. With covariate information and spatial components, models based on Bayesian statistics provide a more accurate estimation of the relative risk of each sub-district. In addition, methods of spatial statistics and analysis were applied in this study to identify and map spatial patterns.

Another important topic is the analysis scale, which is often known as modifiable areal unit problem [28,29]. The analysis scale includes the size of the units in which phenomenon are measured and the size of the units into which measurements are aggregated for data analysis and mapping [30]. To study a phenomenon accurately, it has been suggested that the analysis scale must match the actual scale of the phenomenon [31]. However, this issue can become quite difficult, especially in unfamiliar cases. Traditionally, geographers analyze phenomena in geographical units that are as small as possible [14,32,33], which results in difficulties and high expenses for the data collection. Furthermore, the choice of the analysis scale is often dictated by the availability of data, and because of sparse data, there will often be a tradeoff between homogeneity within small geographic units and the precision of risk estimates [13,16]. Because of the availability of census data, the study is performed at the sub-district level, even though smaller geographical units existed in the study area.

In this work, we explored the spatial heterogeneity of the relative risk for hypertension admissions throughout Shenzhen in 2011 and attempted to address the drawbacks of the standardized ratio in disease mapping. Spatial statistical techniques and methods based on hierarchical Bayesian models were utilized in this study, and both covariate information and random components were employed in these models. After smoothing the relative risk of hypertension, a stable standardized ratio was acquired in each sub-district to highlight those sub-districts that have elevated or lowered relative risk. Our study aimed to identify some specific regions with high relative risk for hypertension admissions, and this information is useful for the Shenzhen City’s health administrators to improve the quality of hospital-based services for hypertension patients.

## 2. Materials and Methods

### 2.1. Description of the Study Area

**Figure 1:**
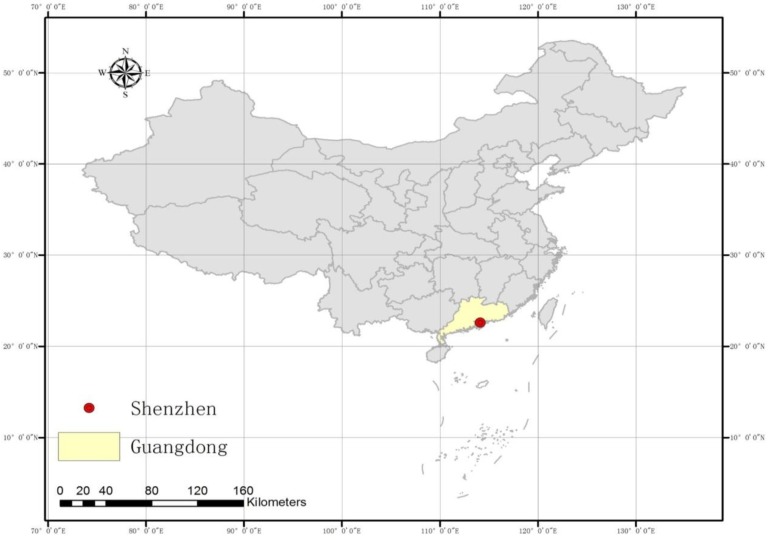
A map of China showing the location of Shenzhen.

**Figure 2:**
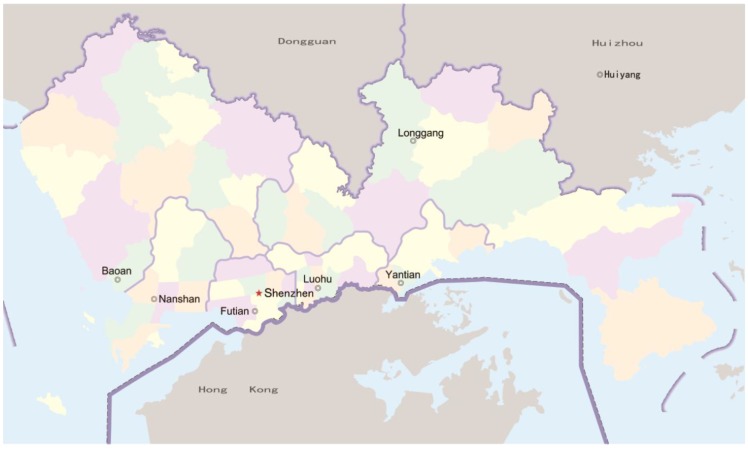
A map of Shenzhen.

Shenzhen is a major city in the south of Southern China’s Guangdong Province, and it is situated immediately north of Hong Kong (Figure 1 and Figure 2). Since late 1979, this area has become one of the most successful Special Economic Zones in China and is considered one of the fastest-growing cities in the World. The total annual investment in medical and health in 2008 was 3.3 billion Yuan, and this investment reached almost 7.9 billion Yuan in 2011 [34].

One of the major challenges for Shenzhen City’s health and medical administration is dealing with the increasing burden from chronic disease accompanied by the population’s fast expanding. According to the population screening report in Shenzhen in 2008, the overall hypertension awareness and treatment rates were low. Moreover, the incidence of hypertension in Shenzhen has doubled during the period from 1997 to 2009 [35]. In addition, the prevalence of overweight and obese children in Shenzhen is not far behind the levels observed in children from Australia, the United Kingdom and the USA [36]. Besides, the hospital admissions rate for chronic disease can be reduced by effective primary and secondary prevention in primary care [37], thus analyzing and estimating the relative risk of hospital admissions for hypertension disease accurately is useful for the health administrators to develop a high-quality and well-organized hospital-based health services for hypertension patients.

The other subject that interests us is the unique characteristics of Shenzhen. In a relatively short period of 34 years, Shenzhen has become a thriving city with a modern cityscape, which is distinctive in the world. Because of its rapid economic growth and high population density, Shenzhen is a typical urbanized area in China. It has important significance to analyze the spatial variation of hypertension admissions with Shenzhen’s fast-paced style for health administration and disease control.

### 2.2. Data

Since 2006, the Shenzhen Municipal Government began to create and apply a universal urban spatial grid; it was completed at the beginning of 2010. The spatial grid pyramid contains a multilevel spatial grid, namely, the city level, district level, sub-district level, community level and basic grid level; 57 sub-districts are included in this grid pyramid. In this study, we employed spatial grids at the sub-district level as the analysis units and the data were aggregated into this level.

Hypertension data on the total number of hospitalized patients in each sub-district by age, gender and other information were provided by Shenzhen Center for Health Information, which contained 10,419 cases of hypertension in 2011. ICD-10 is the 10th revisions of the International Statistical Classification of Diseases and Related Health Problem (ICD), a medical classification list by the WHO. It codes for diseases, signs and symptoms, abnormal findings, complaints, social circumstances, and external causes of injury or diseases [38]. In our study, we used the data for hypertension (ICD-10 I10-I15), containing primary hypertension, hypertensive heart disease, hypertensive renal disease, hypertensive heart disease and hypertensive renal disease, and secondary hypertension. We aimed at this current study to examine the regional changes in the hospital admissions for hypertension disease, not to explore the age/sex disparities in hypertension. Thus, the further differentiations of these cases were not implemented because modeling the relationships between the population’s age and sex composition and the hospitalization rates of hypertension was not included in this research.

Based on the data from the Sixth National Population Census, the population data were aggregated at the sub-district level. Although the census was carried out in 2010 and may not match the actual distribution of the potential hypertensive population in 2011 perfectly, it can still be applied for estimating the standardized ratio because of the age and mental stress-orientation of hypertension and the region’s demographic stability. Previous works have demonstrated that being overweight and having hypertension are more prevalent in the more urbanized areas [39], and the road density is a common way of quantifying urbanization [40]. Hence, the road density of each sub-district was employed as a covariate in the model, which was calculated by dividing the total length of the roads of each sub-district by its area.

### 2.3. Standardized Ratio Calculation

The standardized ratio, which is expressed as a ratio or percentage of observed cases count in the study area to the expected cases count in the general population, is used to determine if the occurrence of a disease in a relatively small population is high or low. Because the hypertension data were extracted from the information of hypertension hospitalization cases and local age-specific admission rates were not available, we applied the standardized admission ratio in each sub-district as the standardized ratio, which can be used to represent the relative risk for hypertension admission across the study area. In this study, the number of expected cases of hypertension admission was calculated by multiplying hypertension admission rate of the general population by the resident population of each sub-district. Thus, this kind of standardized ratio was defined based on indirect standardization method and calculated as follows:

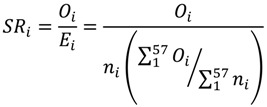
(1)
where *O_i_* and *E_i_* denotes the observed and expected counts of hypertension admission cases in sub-district “*i*” respectively, and *n_i_* represents the resident population of each sub-district “*i*”.

### 2.4. Bayesian Model-Based Disease Mapping

To overcome the drawbacks of the standardized ratio, models based on Bayesian statistics are widely adopted in disease mapping. We assumed that the observed hypertension hospitalization cases (*O_i_*) at the sub-district level followed a Poisson distribution with mean *µ_i_*. In addition, *µ_i_* is an estimate of the true number of hypertension hospitalization cases in sub-district “*i*”, which can be provided by the expected hospitalization cases in sub-district “*i*” (*E_i_*) and the standardized ratio (*SR_i_*) in sub-district “*i*”. A general formulation is given by:

*O_i_ ~ Poisson* (*µ_i_*) (2)

*E* (*O_i_*) = *µ_i_ = E_i_ × SR_i_* (3)

In Poisson-distributed models, the standardized ratio in sub-district “*i*” can be explained by a series of explanatory variables. With an intermediate distribution of the logarithm of the standardized ratio, this model can be parameterized as:

log(*SR_i_*) = *α*_0_ + *βX +*ϵ*_i_* (4)

In Equation (4), the log of the standardized ratio in each spatial unit “*i*” is modeled by an intercept term *α*_0_, a series of explanation variables constituted by a set of covariates *X* and regression coefficients *β* and ϵ*_i_*, which is interpreted as the residual term results from unknown or unobserved factors. Although the residual term is assumed to be approximately normally distributed, there are two sources of variability that may appear in disease mapping studies at the area level that will violate this statistical assumption. In tackling this problem, the residual term can be divided into two segments: a spatially correlated (structured) component and a spatially random (unstructured) component that represents the spatial correlation and overdispersion in the residual term of each sub-district. Therefore, the model is revised as follows:

log(*SR_i_*) = *α*_0_ + *βX + U_i_ + S_i_* (5)

log(*µ_i_*) = log(*E_i_*) + *α*_0_ + *βX + U_i_ + S_i_* (6)

*α*_0_ ~ *U*(*− ∞, + ∞)* (7)

*β ~ N* (0, *τ*^2^) (8)

*U_i_ ~ N* (0, *δ*^2^) (9)

*S_i_ ~ CAR*(*σ*^2^) (10)

In Equation (5), *U_i_* denotes the unstructured random component and *S_i_* represents the structured random component of each sub-district “*i*”. When modeling based on a Bayesian framework, it is necessary to specify a prior distribution for the observed data. A noninformative prior distribution (flat distribution) or weakly informative Gaussian prior distribution (a normal distribution with large variance) could be given for the priors of the intercept term and covariate coefficients in Equations (7) and (8) [15,41]. In Equation (9), the unstructured component was assigned to follow a normal distribution with mean zero and variance *δ*^2^, and the spatially correlated component was introduced through a conditional autoregressive prior distribution (CAR) with variance *σ*^2^ in Equation (10) [42,43], which is a type of Markov random field model. We applied a robust version of CAR in this research that assumed a double exponential distribution rather than an intrinsic Gaussian CAR prior distribution [44].

Specifying suitable priors for the variance of the unstructured and structured spatial component is another critical subject because the differences between the sizes of the priors for *σ*^2^ and *δ*^2^ could result in a disparity in spatial smoothing. A useful approach is to assign a prior to the standard deviation rather than to the precision that is the reciprocal of the variance. Gelman recommended using a uniform prior distribution with a wide range instead of the inverse-gamma family of noninformative prior distributions for the hierarchical standard deviation [27]. For the standard deviation, we set a uniform distribution on the interval (0,100) because this range was wide enough to cover any realistic value for the standard deviation in log-transformed modeling.

These models were coded in the WinBUGS 1.4 software [45], which could be called from R with R2WinBUGS. WinBUGS (Bayesian inference Using Gibbs Sampling from R) was designed as flexible software for the Bayesian analysis of complex statistical models using Markov chain Monte Carlo (MCMC) methods [44]. For each model, three parallel MCMC chains that each of 20,000 MCMC iterations were simulated and visualized with time series plots and Gelman-Rubin statistics [15]. Then, the posterior distribution of the smoothing standardized ratio was acquired after a burn-in of 2000 iterations. The deviance information criterion (DIC) [46] was utilized to compare all of the models to determine the “best fit” model. The DIC is a hierarchical modeling generalization of the Akaike information criterion (AIC) and Bayesian information criterion (BIC); it is particularly helpful in a Bayesian model selection problem where the posterior distributions of the models are acquired based on an MCMC simulation. The generalization is based on the posterior distribution of the deviance statistic that is defined as follows:

*D*(*θ*) = −2*logf* (*yǀθ*) + 2log *h*(*y*) (11)

where *f* (*yǀθ*) is the likelihood function and *h*(*y*) is some standardizing function of the data alone which is a constant that cancels out in all calculations that compare different models. In [46], the authors suggest summarizing the fit of a model by the posterior expectation of the deviance, given by *D = E_𝜃_*_ǀ_*_y_*[*D*]. Then, *pD* is the effective number of parameters to measure the complexity of a model; however *pD* may well be less than the total number of model parameters, due to the borrowing of strength across random effects . A reasonable definition of *pD* is the posterior expected deviance minus the deviance evaluated at the posterior expectations [24]. Thus, *pD* is given by:

*pD= E_𝜃ǀy_*[*D*] - *D* (*E_𝜃__ǀy_*[*θ*]) = *D - D* (*θ*) (12)

Using the notation in the WinBUGS output for the DIC tool, *Dbar* represents the posterior expected deviance and *Dhat* denotes the deviance evaluated at the posterior expectations. Then, *pD* and DIC is given by

*pD = Dbar - Dhat* (13)

*pD = E_𝜃ǀy_*[*D*] - *D* (*E_𝜃ǀ__y_*[*θ*]) = *D - D* (*θ*) (14)

*DIC = Dbar + pD = Dhat +* 2*pD* (15)

## 3. Results

### 3.1. The Spatial Variations of the Observed Admission Cases at Multiple Levels

**Figure 3:**
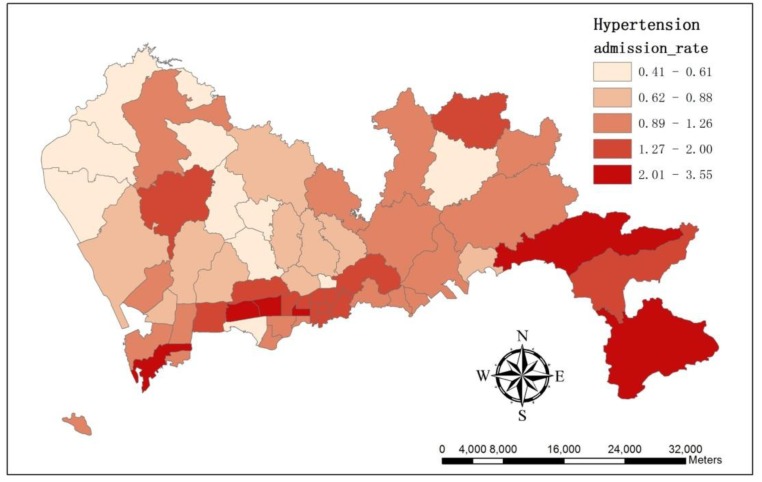
The map illustrates the spatial variation of hypertension admission rate at sub-district level.

We applied the number of hypertension admission cases per 1,000 people in each sub-district as the admission rate. The result showed hypertension admission rate varied across the study area (Figure 3). The sub-district *Lianhua* suffered the highest admission rate as 3.55, whereas in *Longhua*, the rate was only 0.41.

**Figure 4:**
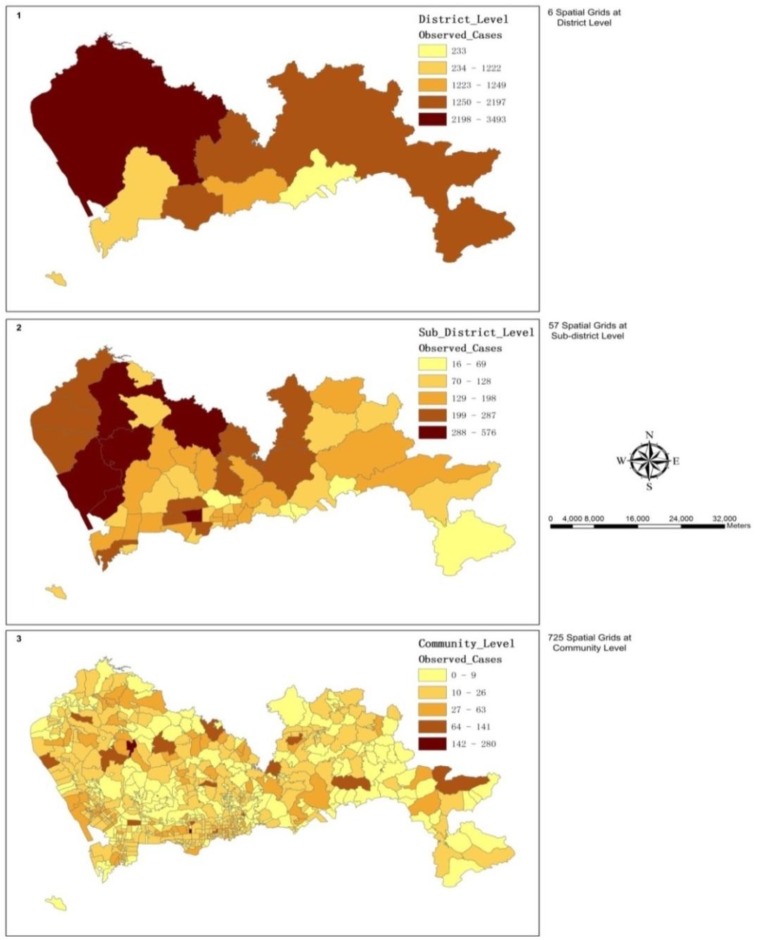
These maps illustrate the spatial variations of the observed hypertension admission cases at multiple levels: (**1**) the district level; (**2**) the sub-district level, and (**3**) the community level.

The observed hypertension admission cases varied across the study area, and the spatial patterns were dissimilar at multiple analysis scales (Figure 4). Moran’s Index (Moran’s *I*) was applied in this research to identify and measure the strength of the spatial patterns of the observed hospitalization cases in neighboring sub-districts. The results showed there was a statistically significant cluster pattern in the observed cases count of nearby sub-districts with a *p*-value < 0.01. Then, the Getis-Ord General G-statistic was adopted to measure the concentration of the values of the observed cases count at the sub-district level. With a large *z*-score of 3.65, this statistics indicated that the spatial distribution of the high values of the observed cases count was spatially clustered.

### 3.2. The Spatial Variation of the Relative Risk of Hospital Admissions for Hypertension

**Figure 5:**
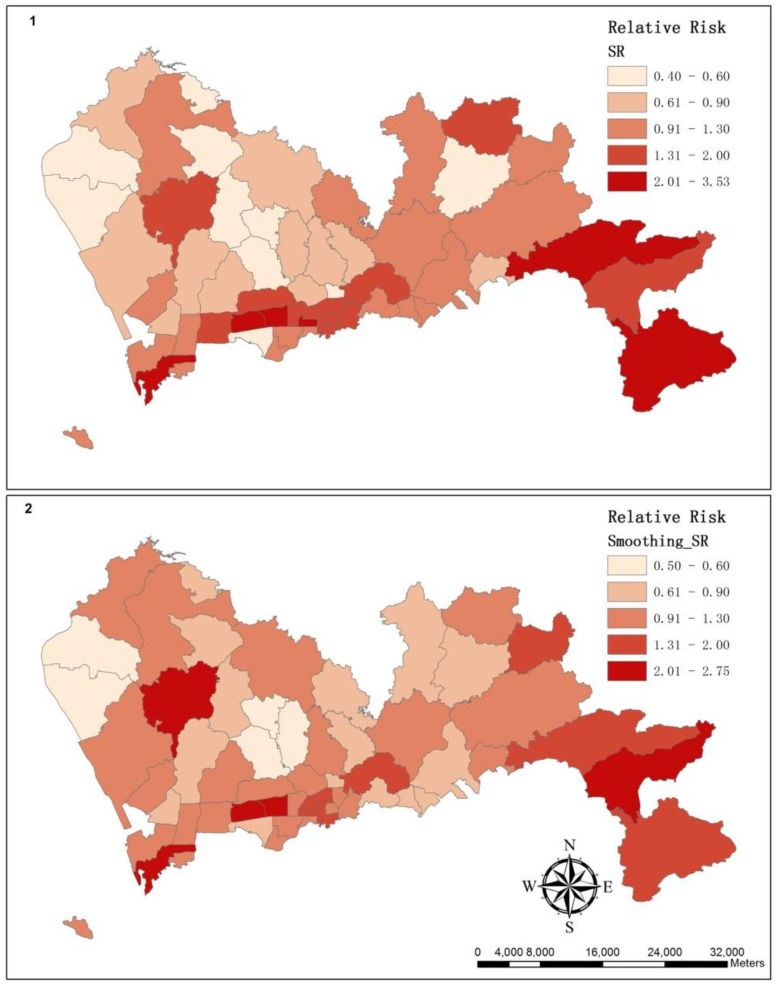
These maps illustrate the spatial variation of relative risk: (**1**) a map of the SR; (**2**) a map of the smoothing SR.

**Figure 6:**
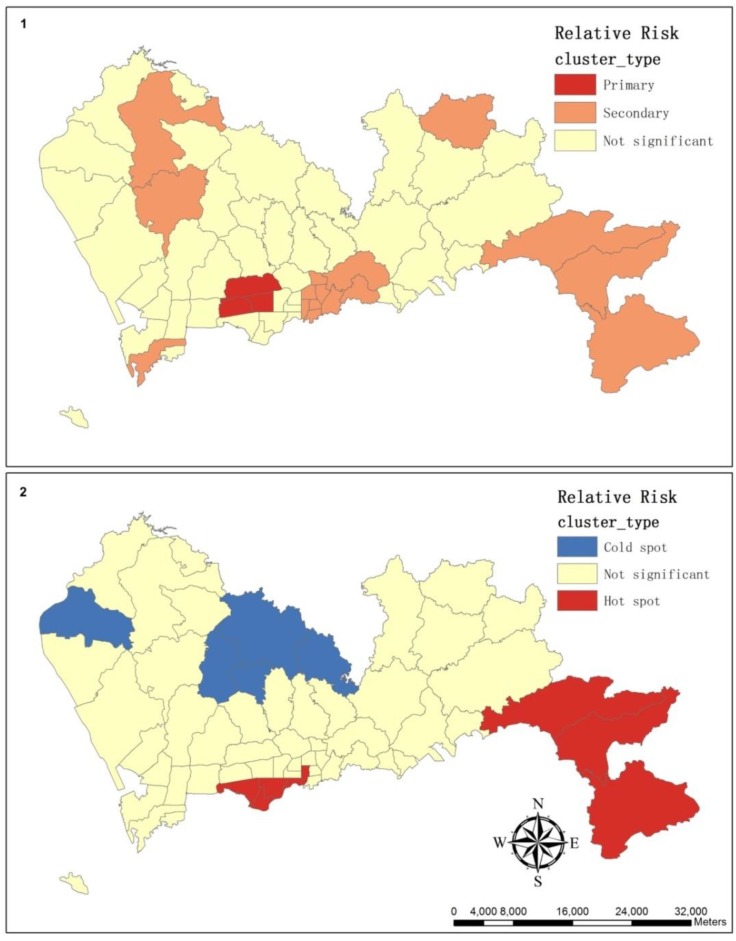
These maps illustrate the cluster of relative risk as estimated by SatScan (**1**) and as estimated by the hot spot analysis of ArcGIS (**2**).

The map of the SR illustrates that the relative risk varied throughout Shenzhen (Figure 5(1)). The sub-district *Lianhua* had the highest relative risk with an SR of 3.53, whereas in *Longhua*, this risk was only 0.40. The results of Moran’s *I* showed that the cluster pattern was statistically significant in the standardized ratios of adjacent sub-districts with a *p*-value < 0.01 and a z-score of 2.60. By using the General G-statistic, a high-value cluster was significant with a z-score of 2.46. Then, a hot spot analysis based on the local G-statistic (Gi*) was used to show where the clusters of high values or low values were, and results were summarized in Table 1. A group of sub-districts with high Gi* values indicated a concentration of sub-districts with a high SR as a hot spot; conversely, a group of sub-districts with low Gi* values indicated a cold spot (Figure 6(2)).

**Table 1:** The results of hot spot analysis based on the Gi*.

Cluster Type	Sub-district	Observed Cases	Expected Cases	SR	GiPValue	GiZscore
**Hot spot**	*Fubao*	107	107.21	1.00	0.01	2.55
	*Futian*	248	247.83	1.00	0.08	1.74
	*Nanyuan*	109	113.97	0.96	0.08	1.77
	*Shatou*	134	226.66	0.59	0.06	1.90
	*Guiyuan*	152	82.59	1.84	0.07	1.82
	*Kuiyong*	176	61.34	2.87	0.02	2.34
	*Nanao*	51	19.05	2.68	0.03	2.14
	*Dapeng*	87	46.44	1.87	<0.01	3.15
**Cold spot**	*Guannan*	370	453.96	0.82	0.08	−1.77
	*Shajing*	287	531.41	0.54	0.09	−1.72
	*Dalang*	147	279.45	0.53	0.05	−1.94
	*Longhua*	148	366.27	0.40	0.02	−2.39
	*Pinghu*	219	229.34	0.95	0.06	−1.91

SaTScan is a free software package that analyzes spatial, temporal and space-time data using the spatial scan statistics, which are widely used in performing geographical surveillance of disease and disease-detecting clusters and in testing whether a disease is randomly distributed over space, over time or over both space and time [47]. According to a known underlying population at risk, a purely spatial analysis was performed using a Poisson-based model where the hypertension cases in a sub-district were assigned to follow a Poisson distribution. The clusters were mapped in Figure 6(1) and summarized in Table 2.

**Table 2:** The clusters of the relative risk in Shenzhen, 2011 from SaTScan using a purely spatial analysis.

Cluster Type	Sub-district	Observed Cases	Expected Cases	Relative Risk	*p*-value
**Primary**	*Meilin*	212	152.13	2.69	<0.0001
	*Lianhua*	576	163.28	2.69	<0.0001
	*Xiangmihu*	216	82.43	2.69	<0.0001
**Secondary**	*Dapeng*	87	46.44	2.52	<0.0001
	*Kuiyong*	176	61.34	2.52	<0.0001
	*Nanao*	51	19.05	2.52	<0.0001
	*Zhaoshang*	224	80.12	2.84	<0.0001
	*Liantang*	82	84.40	1.44	<0.0001
	*Donghu*	166	83.55	1.44	<0.0001
	*Huangbei*	170	112.03	1.44	<0.0001
	*Cuizhu*	160	115.91	1.44	<0.0001
	*Dongxiao*	46	103.72	1.44	<0.0001
	*Dongmen*	153	91.46	1.44	<0.0001
	*Sungang*	94	63.47	1.44	<0.0001
	*Nanhu*	157	90.83	1.44	<0.0001
	*Guiyuan*	152	82.59	1.44	<0.0001
	*Shiyan*	444	248.76	1.82	<0.0001
	*Pingdi*	142	95.70	1.49	0.0014
	*Gongming*	400	320.10	1.26	0.0018

### 3.3. Summary of the Hierarchical Bayesian Models

In measuring the importance of explanation variables in modeling the relative risk of hospital admissions for hypertension across the study area, several models with different combinations of variables were examined in Table 3. All of the models were run in WinBUGS 1.4 via Gibbs sampling. We summarize the models with their levels of complexity, and the results show that the models with smaller DIC values were those with intercepts and random effects. Model ***5***, which had the smallest DIC of 359.153, was selected as the best model; it includes an intercept, a spatially structured component and a spatially unstructured component. Table 4 gives the posterior summary for the precision and explanation variables.

**Table 3:** The results of hierarchical Bayesian models from WinBUGS with different complexities.

# of Model	Description	*Dbar*	*Dhat*	*pD*	*DIC*
***1***	*Intercept & road density with coefficient*	3,015.580	3,013.600	1.985	3,017.570
***2***	*Intercept & road density without coefficient*	3,283.520	3,282.530	0.995	3,284.520
***3***	*Intercept & unstructured component*	328.936	283.227	45.709	374.646
***4***	*Intercept & structured component*	334.725	291.671	43.054	377.779
***5***	*Intercept & unstructured & structured component*	316.465	273.777	42.688	359.153
***6***	*Intercept & road density with coefficient & structured & unstructured component*	356.994	306.799	50.195	407.189

**Table 4:** A posterior summary of the results of hierarchical Bayesian models from WinBUGS.

# of Model	Explanation Variables	Mean	SD	MC Error	Credible Interval	
					2.5%	97.5%
***1***	*Intercept*	−0.2729	0.02323	2.873E-4	−0.3187	−0.228
	*Coefficient*	0.4525	0.03394	4.168E-4	0.3862	0.519
***2***	*Intercept*	−0.6257	0.009773	3.893E-5	−0.6449	−0.6066
***3***	*Intercept*	0.05549	0.07219	0.001014	−0.08567	0.197
	*Variance of unstructured component*	4.373	1.032	0.006073	2.633	6.666
***4***	*Intercept*	0.06246	0.03228	1.39E-4	−0.001213	0.1252
	*Variance of structured component*	1.015	0.1706	0.001032	0.7188	1.386
***5***	*Intercept*	0.07391	0.05503	5.591E-4	−0.03969	0.1805
	*Variance of unstructured component*	819.7	13,800.0	435.8	3.965	1,710.0
	*Variance of structured component*	2.207	2.364	0.08871	0.9082	6.592
***6***	*Intercept*	−0.03228	0.2274	0.01064	−0.4436	0.4754
	*Coefficient*	0.1787	0.3556	0.0167	−0.6419	0.8033
	*Variance of structured component*	17.75	203.0	7.739	0.8435	48.67
	*Variance of unstructured component*	26.78	188.6	6.437	3.147	122.8

With the smallest DIC, model ***5*** was selected as the best model that can be used to smooth the standardized ratio that was displayed by the choropleth map in Figure 5(2). By utilizing Moran’s *I*, the cluster pattern was significant in smoothing standardized ratios of neighboring sub-districts with a *p*-value less than 0.05 and a z-score of 2.49. Then, the results of the General G-statistic implied that the highly clustered pattern was significant with a z-score of 2.82.

## 4. Discussion and Conclusions

In recent years, researchers have applied statistical techniques and spatial analysis to study the spatial variation of hospital admissions for hypertension disease. To that end, a Local Moran’s I index analysis and geographically weighted regression were used to investigate the patterns in standardized ratios of cardiovascular disease [48]; furthermore, the risk factors for hypertension was examined using Kaplan-Meier methods and Cox proportion hazards models [49]; in addition, the spatial scan statistics were used to detect clusters of high or low prevalence of overweight people or people with hypertension in rural South Africa [50]. In this study, spatial scan statistics from SaTScan 9.1 were utilized to spot clusters of the relative risk of hospital admissions for hypertension in Shenzhen, spatial statistical techniques from ArcGIS 10.0 were adopted to identify patterns in the standardized ratio and methods based on Bayesian statistics were used to smooth the relative risk in a small-area disease mapping.

Moran’s I index compares the value for each feature in the pair to the mean value for the dataset rather than directly comparing the attribute values off neighboring features to each other. If the average difference between neighboring features is less than the average between all features, the values of the features are clustered. The results of Moran’s I index demonstrated that the spatial autocorrelation was positive in this geographical context, and the spatial dependence of nearby observed cases should be included in the modeling of estimates of the relative risk for hypertension admissions. Then, the Getis-Ord General G-statistic was used in this study to measure the concentration of values. One of the disadvantages of the Getis-Ord General G-statistic is that the results are highly dependent on the size of the features being analyzed. When large areas tend to have low values and smaller areas tend to have high values, even if the concentrations of highs and lows are equally distributed, the G-statistics will indicate that the high values are concentrated. Because the study area is often divided based on the population size and is delineated by the administrative boundaries, this tendency is especially significant when studying geographical phenomena and will lead to bias in analyzing and mapping the high-value clustering.

In hot spot analysis, we applied the Gi* statistic because it included the value of the target feature that affected the occurrence of the clusters. Apart from the hot spot and cold spot, the Gi* values of the rest of the areas were not statistically significant, which means there was no apparent concentration of either high or low standardized ratio surrounding these areas, and this usually happened either when the surrounding standardized ratio was near the mean or when the target sub-district was surrounded by a combination of high and low standardized ratios. The local statistic works best for identifying high-value clusters when there is no measureable pattern of clustering or dispersion across the study area [51].

Then, the spatial scan statistics were applied to spot clusters of the relative risk. The geometry of the area being scanned, the probability-distribution-generating events under the null hypothesis, and the shapes and sizes of the scanning window are the three basic properties of the scan statistic [52]. The methods of the probability approximations and Monte Carlo-based hypothesis testing are applied in the models of the spatial scan statistics, and the local G-statistic uses a neighborhood based on either adjacent features or a set distance. According to the results of a purely spatial scan analysis, the primary and secondary clusters were statistically significant. However, Zhang noted that spatial scan statistics and the local statistic can neither directly incorporate ecological covariates nor account for overdispersion [53].

To tackle the spatial dependence and overdispersion of the standardized ratios, hierarchical Bayesian models were applied in this study. In these models, the standardized ratio was smoothed locally towards the mean ratio in the set of adjacent sub-districts. In small-area disease mapping, estimations of the relative risk are often inaccurate because the population is usually small in the analysis unit. From Table 5, it is clear that the smoothing was greater for the least-stable estimates where the expected number of cases was small. Further research should be conducted in these areas because the larger areas tend to dominate the map visually, even though they produce the least-precise risk estimates.

Although multilevel spatial grids were obtainable, the study was performed at the sub-district level because the available census data were aggregated at this level. However, a disadvantage is that the analysis scales used in many geographical studies are arbitrary and modifiable. For example, the census data may be aggregated into sub-districts, postcode areas, police precincts or any other spatial partition, which affects the analysis’s results. The pattern created by a set of features and attributes may change depending on the scale. Because of the availability of data and the restrictions of research funding, in our study, we specified the sub-district spatial grid as the analysis scale as this scale was capable of describing the spatial variation of the relative risk of hospital admissions for hypertension in Shenzhen. The high-value cluster pattern was statistically significant in the observed cases count and in the relative risk in the neighboring sub-districts, which indicated that the spatial autocorrelation was positive and that the spatial dependence should be included in modeling the relative risk. One of the major contributions of this study was highlighting those sub-districts where the relative risk of hospital admissions for hypertension was concentrated, and the improvement of public health services should be addressed in these areas. Further work on data collection should target the smaller geographical units.

**Table 5:** A summary of the top ten sub-districts with significant smoothing; the rank is specified from high to low.

Sub-district	SR	Smoothing SR	Rank of Expected Cases	Rank of Area
*Kuiyong*	2.87	1.36	53	5
*Huaqiangbei*	2.73	1.78	51	54
*Nanao*	2.68	1.82	56	2
*Lianhua*	3.53	2.75	25	43
*Shahe*	1.62	0.94	30	34
*Pingdi*	1.48	0.91	39	16
*Yantian*	1.19	0.69	47	18
*Donghu*	1.99	1.51	44	25
*Dongmen*	1.67	1.20	40	57
*Shatoujiao*	1.14	0.71	54	45

Another disadvantage of our method is the boundaries of sub-districts, which are a reflection of administrative needs rather than the actual spatial distribution of epidemiological factors [14]. As a result, these boundaries can lead to an inaccurate interpretation of the spatial variation of the relative risk across the study area. Furthermore, the study area is delineated by these artificial boundaries: the realistic process continues beyond the area because it has an interaction with the neighbors outside these borders. Because the calculations are usually based on the spatial neighborhood around each feature, certain spatial statistical techniques may require data on variables that refer to spatial units beyond the boundary of the study area. If these boundary data are not available, this shortcoming represents a form of data incompleteness [16] unless there are fixed natural barriers that would minimize any influence from the surrounding features, as in the case of an island where the coastal boundary affects the spread of some diseases. How the boundary is handled and how to define spatial neighborhoods and weights is a hot topic in spatial analysis and statistics. Some solutions to this problem have been proposed in previous works [16,54]. However, regardless of how the boundary is defined, the features near the edge of the study area will still have fewer neighbors than the features in the center of the study area. In our study, we concluded that the boundary’s effect on the Gi* statistic did not lead to an underestimate problem because each hot spot or cold spot was identified by comparing the local sum to the expected local sum, and furthermore, the differences in the number of neighbors will not impact the result.

We attempted to identify those factors that are associated with the spatial distribution of hospital admissions for hypertension. Previous works have demonstrated the relationship between the prevalence of hypertension, and socioeconomic measures, environmental variables and neighborhood characteristics [5,6,12,21,40,49,55]. In [56], the researchers aimed to investigate the association of aircraft noise with risk of chronic diseases in the general population and concluded that high levels of aircraft noise also associated with an increased risk of stroke, coronary heart disease and cardiovascular disease. In [57], the objective was to investigate whether exposure to aircraft noise increases the risk of hospitalization for cardiovascular diseases in older people residing near airports and the results showed that there was a statistically significant association between exposure to aircraft noise and risk of hospitalization for cardiovascular diseases among older people living near airport. In our study, the results revealed that the road density, which was an indirect factor, can be applied in modeling the spatial variations of the relative risk. In [58], the results indicated that there were ethnic differences in clinical trials and in routine care for diabetes patients in South Asian. Thus, detailed information is necessary in a geographic correlation study, which is usually conducted at a more local or small-area scale, resulting in a demand of large amounts of data. In acquiring the traffic noise level in each neighborhood as a direct factor, noise-dispersion models and manual noise assessments could be used [55]. 

The results of the hierarchical Bayesian model showed that the relative risk of hospital admissions for hypertension was not homogeneous throughout Shenzhen. The high-value clustering was significant in the south and southeast of Shenzhen, which can be applied as a guideline for the establishment of hospital-based health services. However, there was an obvious underestimation in this study because of the lack of awareness of hypertension. Furthermore, Shenzhen is not yet facing a serious aging problem compared with other large Chinese cities, and hence, Shenzhen has a relatively low prevalence of hypertension and admission rate. In addition, the census data suffer drawbacks that result from the policy of “Hukou” (residents who hold a formal household registration in Shenzhen), and the population of Shenzhen is unique because the majority of its residents are migrant workers. Because there is a strong connection between this population group and their hometowns and families, they tend to support the elderly, who may suffer hypertension, which increases the number of hypertension admission cases. The main objective of this study was to improve the estimates of the spatial variation of the relative risk and identify a hot spot for public health services. Further work is necessary to amend the model and explain the spatial heterogeneity of the relative risk that is explored in this study.
